# Clinical outcomes of previously untreated patients with unresectable intrahepatic cholangiocarcinoma following proton beam therapy

**DOI:** 10.1186/s13014-019-1451-5

**Published:** 2019-12-27

**Authors:** Shosei Shimizu, Toshiyuki Okumura, Yoshiko Oshiro, Nobuyoshi Fukumitsu, Kuniaki Fukuda, Kazunori Ishige, Naoyuki Hasegawa, Haruko Numajiri, Keiko Murofushi, Kayoko Ohnishi, Masashi Mizumoto, Tetsuo Nonaka, Hitoshi Ishikawa, Hideyuki Sakurai

**Affiliations:** 1 Department of Radiation Oncology and Proton Medical Research Center, Faculty of Medicine, University of Tsukuba, 1-1-1, Tennodai, Tsukuba, Ibaraki 305-8575 Japan; 2 Department of Gastroenterology, Faculty of Medicine, University of Tsukuba, Tsukuba, Ibaraki Japan

**Keywords:** Intrahepatic cholangiocarcinoma, Proton beam therapy, Survival, Macroscopic type, Unresectable ICC

## Abstract

**Background:**

The effectiveness of proton beam therapy (PBT) as initial treatment for patients with unresectable intrahepatic cholangiocarcinoma (ICC) is unclear, particularly as related to ICC histological subtypes. We performed this study to address this gap in knowledge.

**Methods:**

Thirty-seven patients with unresectable ICC who underwent PBT as their initial treatment were evaluated. Twenty-seven patients had Child-Pugh class A liver function, 11 exhibited jaundice, and 10 had multiple tumors. Nineteen, 7, and 11 tumors were classified as mass forming (MF), periductal infiltrating (PI), and intraductal growth (IG) types, respectively, based on gross appearance in imaging studies. Patients were classified into the curative group (*n* = 25) and palliative group (*n* = 12) depending on whether the planning target volume covered all the macroscopic tumors.

**Results:**

The 1- and 2-year overall survival rates were 60.3, and 41.4%, respectively; the median survival time (MST) was 15 months for all patients. The MSTs for curative and palliative groups were 25 and 7 months, respectively. Curative treatment and adjuvant chemotherapy significantly improved overall survival, while the presence of periductal infiltrating type tumors was a negative prognostic factor. In the curative group, the 1- and 2-year local control rates were 100 and 71.5%, respectively, while the 1-, and 2-year progression-free survival rates were 58.5, and 37.6%, respectively. No severe acute toxicities were observed. Three patients experienced grade 3 biliary tract infection, although it was unclear whether this was radiotherapy-related.

**Conclusion:**

PBT may yield to improve survival and local tumor control among patients with unresectable ICC.

## Precis

Proton beam therapy as the initial treatment significantly improves survival in patients with intrahepatic cholangiocarcinoma, and has a good safety profile. This is especially true when administered as curative treatment in combination with chemotherapy.

## Introduction

Intrahepatic cholangiocarcinoma (ICC) is the second most common primary liver cancer by hepatocellular carcinoma and accounts for 10% of primary liver malignancies. Although surgery is considered the only curative treatment for ICC, only 30% of such tumors are resectable at the time of diagnosis. The standard treatment for unresectable ICCs is chemotherapy; however, the median survival time (MST) for such patients is dismal [1]. Until recently, radiotherapy was indicated for intrahepatic malignancies only as a palliative treatment; however, recent technological advances have allowed for the use of radiotherapy as curative-intent treatment, with favorable results even observed in patients with ICC [2, 3]. In particular, proton beams can achieve excellent dose localization, and we previously reported favorable outcomes in patients who underwent proton beam therapy (PBT) for hepatic tumors including hepatocellular carcinoma (HCC) [4–6], metastatic liver tumors [7, 8], and ICC (including in 20 patients treated between 1995 and 2009) [2].

The gross appearance of ICC is classified into 3 subtypes according to the Liver Cancer Study Group of Japan [9]; the mass forming (MF), periductal infiltrating (PI), and intraductal growth (IG) types. These 3 tumor subtypes have different biological behaviors and are associated with different prognoses after surgical resection. It is generally considered that lymph node metastasis is less frequent in IG-type ICCs [10–12], while perineural invasion is high in MF and PI types [10]. However, the influence of the macroscopic type on the results of radiotherapy has not been previously described. In this study, we analyzed the outcomes of patients who underwent PBT as an initial treatment for unresectable ICC.

## Methods

### Patients

Forty-two patients with ICC were administered PBT as their initial treatment at our current facility between 2001 and 2017. Five of these patients had resectable disease but refused to undergo surgery; of remaining 37 patients, 5, 22, and 10 had unresectable tumors owing to their medical condition (old age or poor performance status [PS]), tumor progression, and both, respectively. The latter 37 patients were investigated in our study. The patients’ characteristics are shown in Table 1; they included 22 men and 15 women with a median age of 68.4 years (range: 32–87 years). The Eastern Cooperative Oncology Group PS scores were 0, 1, and 2 for 12, 19, and 6 patients, respectively. In terms of liver function, 27 and 10 patients were classified as Child-Pugh A and B, respectively. Eleven patients were jaundiced at presentation; 4, 4, 19, and 10 were diagnosed with stage I, II, IVA, and IVB disease, respectively, acording to the TNM classification (UICC version 7). Twenty patients had ICC confirmed via histology while the remainder were diagnosed based on imaging study including dynamic contrast-enhancement CT scans and MRI, positive tumor markers of carcinoembryonic antigen (CEA) or carbohydrate antigen 19–9 (CA19–9) with negative HCC-specific tumor markers such as alpha-fetoprotein (AFP) and protein induced by vitamin K absence or antagonist-II (PIVKA-II). The tumor diameters ranged from 15 to 140 mm with a median of 57 mm. Twenty-seven patients had a solitary tumor and 10 patients had multiple tumors. The tumors in 19, 7, and 11 patients were classified as MF, PI, and IG types, respectively, based on CT findings. 25 and 12 patients received PBT with curative and palliative intent, respectively, depending on whether the planning target volume covered all detected macroscopic tumors including positive lymph nodes (curative) or not (palliative). Five patients of the 10 with multiple tumors received PBT in curative intent but other 5 patients had multiple tumor located at both lobe and lymph node or distant metastases, and they received PBT in palliative intent in order to avoid bile duct stenosis at hepatic portal region. In the curative group that comprised 25 patients, the tumors of 13, 4, and 8 were classified as MF, PI and IG types, respectively.

**Table 1:** Patient Characteristics (*n* = 37)

		Curative group(*n* = 25)	Palliative group(*n* = 12)
Median Age (range)	68.4 (32–87)	72 (44–82)	67 (32–87)
Gender			
Male	22	17	5
Female	15	8	7
Performance status			
0	12	11	1
1	19	13	6
2	6	1	5
3	0	0	0
Child-Pugh Classification			
A	27	20	7
B	27	5	5
Jaundice			
yes	26	21	5
no	11	4	7
Treatment intent			
Curative	25		
Palliative	12		
Median size of the tumor (range) (mm)	57 (15–140)	44 (15–140)	60 (22–110)
Number of tumor			
single	27	22	5
multiple	10	3	7
TNM stage (7th UICC)			
I	4	4	0
II	4	3	1
III	0	0	0
IVa	19	15	4
IVb	10	3	7
T stage			
1	5	5	0
2a	6	4	2
2b	5	1	4
3	1	1	0
4	20	14	6
N stage			
0	21	17	4
1	16	8	8
M stage			
0	27	22	5
1	10	3	7
macroscopic subtype			
Mass forming (MF) type	19	13	6
Intraductal growth (IG) type	7	4	3
Periductal infiltrating (PI) type	11	8	3
Total dose			
66.0 GyE in 10 Fraction (BED*:109.6 Gy)	1	1	0
72.6 GyE in 22 Fraction (BED:96.6 Gy)	21	19	2
74.0 GyE in 37 Fraction (BED:88.8 Gy)	5	2	3
Other (BED < 88.8Gy)	10	3	7
Concurrent chemotherapy			
TS-1	15	11	4
Gemcitabine	1	1	0
None	21	13	8
Adjuvant Chemotherapy			
TS-1	10	7	3
Cisplatine + Gemcitabine	5	4	1
Gemcitabine	4	2	2
None	18	11	7

^*^BED: biological effective dose (α/β = 10)

### PBT administration

For treatment planning, fiducial markers were implanted into the normal liver tissue close to the tumor boundary for patient positioning. The patient’s body was immobilized using an individually shaped body cast (ESFORM; Engineering System Co., Matsumoto). CT using 5 mm-thick slices was then performed in the treatment position during the end-expiratory phase with the support of a respiratory-gaiting system. The clinical target volume for the primary lesion was delineated to encompass the gross tumor with 5–10 mm margins in 3 dimensions, and caudal 5 mm margins were added to compensate for any potential hepatic movement. The clinical target volume for nodal lesion was drawn to cover the clinically positive nodes with 5 mm margins if required. Elective nodal irradiation was not performed. Next, 5–10 mm margins were added to define the planning target volume. Treatment beams were delivered via the double scattering mode during the end of the expiratory phase using a respiratory gating system as described previously [13].

The median prescribed dose was 72.6 GyE in 22 fractions; the doses ranged from 46.6 GyE in 12 fractions to 74.0 GyE in 37 fractions. Basically, the dose prescription for curative-intent therapy was dependent on tumor location, and was 72.6GyE in 22 fractions (biological effective dose (α/β = 10) [BED]: 96.6 Gy) for porta hepatis, 74GyE in 37 fractions (BED: 88.8 Gy) for 2 cm from gastrointestinal tract, and 66 GyE in 10 fractions (BED:109.6 Gy) for tumor not adjacent to gastrointestinal tract and porta hepatis. The relative biological effectiveness of the proton beam was designated as 1.1 [14]. The tumor was covered by > 95% of the prescribed dose at the isocenter, however, the target volume was usually modified according to dose constraints for the gastrointestinal tract so as not to exceed a maximum dose of 50 Gy. Also, the percentage volumes of normal liver receiving at least 0, 10, 20, and 30 GyE (V0, 10, 20, and 30) of 30, 20, 26, and 18% were used as an adequate indication [15]. One, 21, and 5 patients received 66.0 Gy in 10 fractions, 72.6 Gy in 22 fractions, and 74.0 Gy in 37 fractions, respectively. 10 patients received less than an BED of 88.8 GyE (equivalent to 74 Gy in 37 fractions): 70.0 Gy in 35fractions, 66.0 Gy in 20 fractions, 66.0Gy in 30 fractions, 59.4 Gy in 18 fractions, 55.0 Gy in 10 fractions, and 46.6 Gy in 12 fractions for 1, 2, 1, 1, 2, and 1 patients, respectively (Table 1).

### Chemotherapy

Sixteen patients received concurrent chemotherapy to achieve a radiosensitizing effect, 15 of whom received oral chemotherapy with tegafur, gimeracil, and oteracil (TS-1), while the remaining patient received intravenous gemcitabine. Maintenance chemotherapy was administered after PBT to 19 patients, 10 of whom received TS-1 and 9 received intravenous chemotherapy (cisplatin plus gemcitabine: 5, gemcitabine alone: 4).

### Analysis

The overall survival rate was calculated from the date of PBT commencement to that of death or March 2018. Local failure was defined as an increase of at least 20% in the sum of the target lesion diameters by diagnostic imaging such as CT and MRI. Progression was defined as local recurrence or the appearance of new legions. Toxicities were graded according to National Cancer Institution Common Terminology Criteria for Adverse Events version 4.0 [16].

Survival and local control rates were calculated using the Kaplan-Meier method, and differences between 2 groups were determined using the log-rank test. Factors that significantly influenced survival and local control were identified using the Cox proportional hazards model. A *P*-value of < 0.05 was considered significant.

## Results

### Survival

**Fig. 1:**
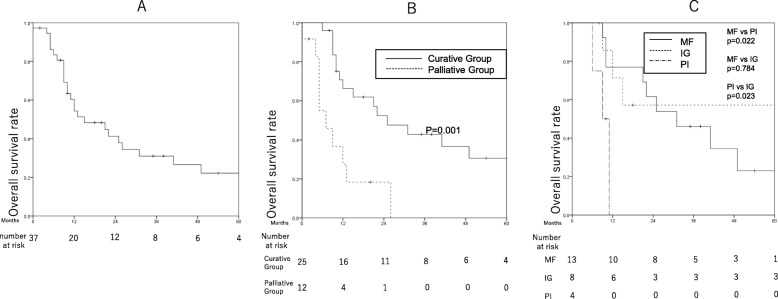
Kaplan-Meier curves showing the overall survival of patients who underwent proton beam therapy as primary treatment for intrahepatic cholangiocarcinoma. **a** Overall survival among all patients. **b** Comparison of overall survival between the curative and palliative groups. (**C**) Comparison of overall survival among curative-group patients with tumors categorized according to the three macroscopic subtypes. MF: mass forming type, IG: intraductal growth type, PI: periductal infiltrating type

Among the 37 patients, 10 were alive at the last follow-up date, 25 had died from cancer progression, and 2 had died from reasons other than ICC. The median follow-up time was 37.5 months, and the MST was 15.0 months with cumulative 1- and 2- years survival rates of 60.3% (95% confidence interval [CI]: 44.7–76.6%), and 41.4% (95% CI: 24.5–58.3%), respectively, for all patients (Fig. 1a). There was a significant difference in survival between the curative and palliative group (*P* = 0.001) (Fig. 1b). In the curative group, the MST was 25 months with 1- and 2-year overall survival rates of 66.3% (95% CI: 47.3–85.3%), and 52.4% (95% CI: 31.8–73.0%), respectively. For patients in the palliative group, the MST was 7 months with 1-, and 2-year overall survival rates of 27.5% (95% CI: 1–54%), and 18.3% (95% CI: 0–41.2%), respectively. Other factors that were associated with significantly improved survival were Child-Pugh A liver function (*P* = 0.004), absence of jaundice (*P* = 0.002), and undergoing maintenance chemotherapy (*P* = 0.000). The MST for patients treated with or without maintenance chemotherapy were 49 months and 10 months, respectively (Table 2).

**Table 2:** Analysis of prognostic factors on overall survival

Foctor		Patient number *n* = 37	MST (months)	Univariate analysis
				*P*-value
Intent	Curative	25	25	0.001
	Palliative	12	7	
Child-Pugh	A	27	25	0.004
	B	10	9	
Jaundice	Yes	11	25	0.002
	No	26	9	
Lymph node metastases	Yes	16	15	0.344
	No	21	12	
Vessel invasion	Yes	32	13	0.558
	No	5	31	
Tumor number	multiple	10	9	0.272
	single	27	21	
Concurrent chemotherapy	Yes	16	41	0.454
	No	21	13	
Adjuvant chemotherapy	Yes	19	49	0.000
	No	18	10	

Although the macroscopic tumor type was not a significant survival factor among all patients combined, it was a significant factor specifically in the curative group, in which survival was significantly worse for patients with PI-type tumors than for those with the other 2 tumor types (PI vs. MF: *P* = 0.022; PI vs. IG: *P* = 0.023; MF vs. IG: *P* = 0.784), and the median survival times were 61 months, 31 months, and 9.0 months for patients with IG-, MF-, and PI-type tumors (Fig. 1c). In the curative group, Child-Pugh classification (*P* = 0.023), jaundice (*P* = 0.005), lymph node metastasis (*P* = 0.032), and maintenance chemotherapy (*P* = 0.000) were also significantly associated with survival.

### Disease progression

**Fig. 2:**
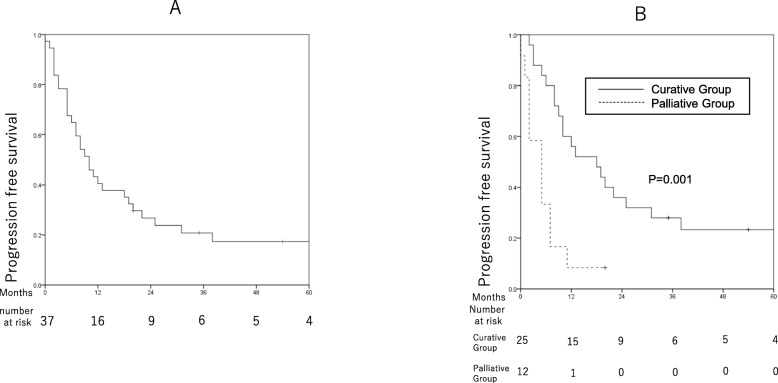
Kaplan-Meier curves showing the progression-free survival of patients who underwent proton beam therapy as primary treatment for intrahepatic cholangiocarcinoma. **a** Progression-free survival among all patients. **b** Comparison of progression-free survival between the curative and palliative groups

The median progression-free survival (PFS) time was 10 months, with 1-, 2-, and 3- years PFS rates of 40.5% (95% CI: 24.6–56.4%), 26.8% (95% CI: 12.5–41.1%), and 25.1 (95% CI: 7.5–34.1%), respectively, for all patients (Fig. 2a). Eight patients were alive without disease progression on the last follow-up date. In the curative group, the median PFS was 19 months with cumulative 1-, 2-, and 3-year PFS rates of 58.5% (95% CI: 38.9–78.1%), 37.6% (95% CI: 18.2–57.0%), and 25.1 (95% CI: 7.6–42.5%), respectively (Fig. 2b). Six of the 25 patients in the curative group were alive; of the remaining 19, the manner of initial disease progression was local progression in 3 patients, intrahepatic recurrence outside the treatment field in 9, extrahepatic recurrence in 2, non-cancer related death in 1, and unknown in 4.

**Fig. 3:**
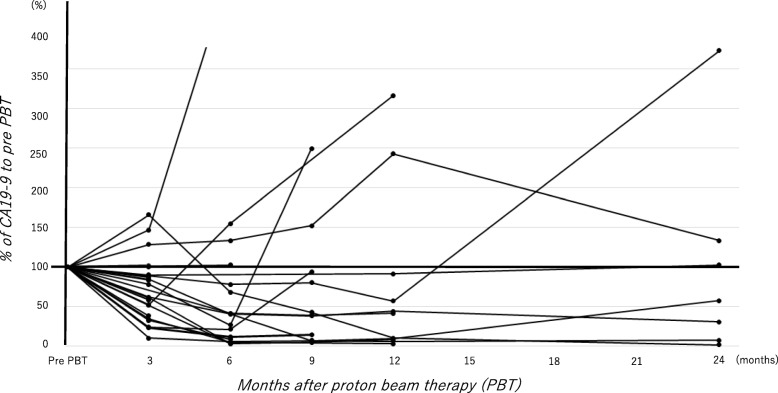
Change of CA19–9 (%) from PBT. Percentage (%) of the value prior to PBT was calculated

The change of CA19–9 was shown in Fig. 3. We calculated percentage (%) of the value prior to PBT. CA19–9 prior to and after PBT was available in 25 patients including 6 patients with palliative intent, and the CA19–9 after PBT decreased in 19 patients of the 25 (76%). Even with palliative intent, CA19–9 was decreased in 4 of the 6 patients. Of the 25 patients, 15 patients received chemotherapy, but CA19–9 was elevated in 5 patients of the 15. Meanwhile, CA19–9 was elevated only one patient in the 10 who did not received chemotherapy. The median % of 3-, 6-, 9-, 12-, and 24- months was 61.6% (9.9–166.0%), 40.5% (2.7–473.9%), 38.7% (4.4–250.0%), 41.5% (2.8–316.6%), and 57.4% (1.8–373.4%), respectively.

### Local control

**Fig. 4:**
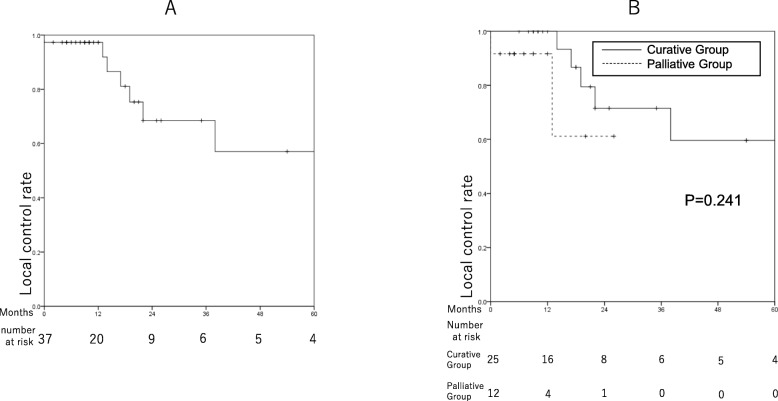
Kaplan-Meier curves showing local control among patients who underwent proton beam therapy as primary treatment for intrahepatic cholangiocarcinoma. **a** Local control rates among all patients. **b** Comparison of local control between the curative and palliative groups

Seven patients experienced in-field local failure. The 1- and 2-year local control rates were 97.3% (95% CI: 92.0–100%) and 68.4% (95% CI: 46.6–90.2%), respectively, for all patients; these rates were 100 and 71.5% (95% CI: 47.6–95.4%), respectively, for curative-group patients (Fig. 4).

Of the 7 patients with in-field local failure, 1 underwent PBT with 46.6 GyE with palliative intent, and the remaining 6 were treated with curative intent. Among patients in the latter group, 1, 3, 1, and 1 received 74 GyE, 72.6 GyE, 70 GyE, and 66 GyE, respectively.

### Toxicity

No severe (grade ≥ 3) acute toxicities were observed. In terms of late adverse events, 3 patients experienced grade 3 biliary tract infections (Table 3) although it was unclear whether these were linked to PBT; all were managed conservatively with antibiotics.

**Table 3:** Toxicities (*n* = 37)

Toxicity	Grade 0 / 1	Grade 2	Grade 3	Grade 4 / 5
Acute				
Dermatitis	36	1	0	0
Gastrointestinal ulcer	34	3	0	0
Late				
Gastrointestinal ulcer	36	1	0	0
Cholangitis	31	3	3	0

### A case presentation

**Fig. 5:**
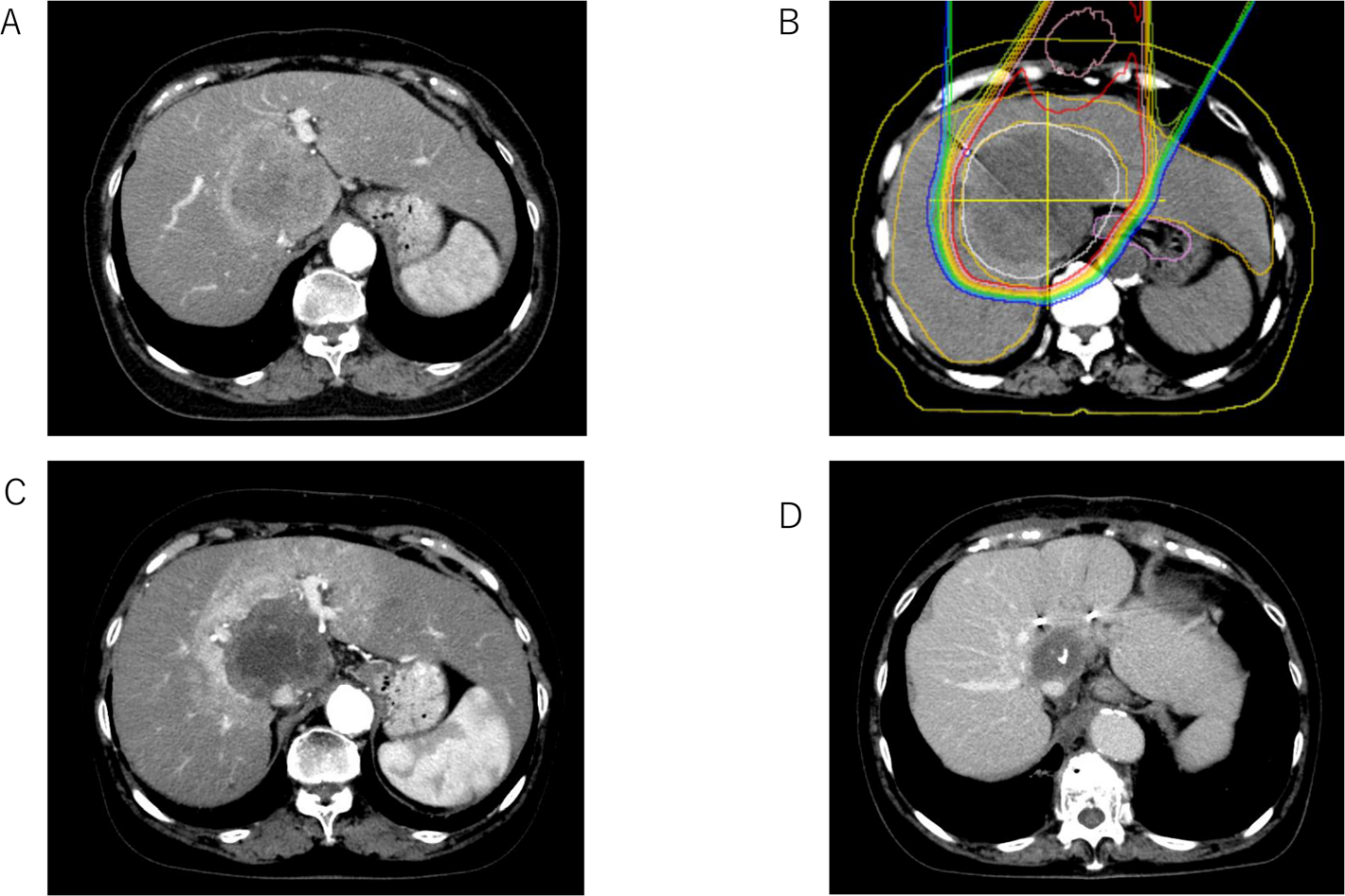
Imaging features of a 75-year-old man with a single intrahepatic 70 mm tumor at the caudate lobe who was diagnosed with intrahepatic cholangiocarcinoma. **a** Computed tomography at diagnosis. **b** The proton beam therapy dose line. **c** Six months after proton beam therapy. **d** Three years after proton beam therapy

A 75-year-old man with a single intrahepatic 70 mm tumor at the caudate lobe was diagnosed with ICC via liver biopsy. Although he had Child-Pugh A liver function, swollen hepatic portal lymph nodes were detected; therefore, we diagnosed this patient with T1N1M0 stage IVA disease. The tumor was enhanced peripherally on CT and therefore classified as MF type (Fig. 5a). Surgery was considered difficult owing to middle- and left-liver vein encasement; therefore, PBT was conducted with 72.6 Gy in 22 fractions (Fig. 5b). Six months after the PBT, the tumor had shrunk although radiation hepatitis was evident within the beam pathway (Fig. 5c). Three years after undergoing PBT, the tumor had shrunk further and the enhanced legion had disappeared; the lymph node had also shrunk. At the last follow-up visit, this patient was alive with no disease progression 4 years after the PBT (Fig. 5d).

## Discussion

The prognosis of patients with cholangiocarcinoma who receive no treatment is much worse. Park et al. investigated 330 patients with cholangiocarcinoma who did not receive surgery, chemotherapy, or radiotherapy; their median overall survival was 3.9 months; moreover, the median survival time of patients in their cohort with ICC (3 months) was significantly worse than that of patients with hilar cholangiocarcinoma (5.9 months, *P* < 0.001) [17]. Chemotherapy for unresectable ICC has been established. Gemcitabine is a major drug used to treat unresectable biliary duct cancer [18–20], and TS-1 is also used as an alternative [21, 22]. However, treatment outcomes are still extremely poor. A large study by Valle et al. of patients with biliary tract cancer suggested that combination cisplatin plus gemcitabine provided a survival advantage over gemcitabine alone, but the median overall was only 11.7 months [18].

Previously, radiotherapy for ICC was limited to palliative treatment because the liver cannot tolerate extensive irradiation [23]. However, the recent advances in radiotherapy, including stereotactic body radiotherapy (SBRT), intensity modulated radiotherapy, and particle treatment has made possible the delivery of high doses to the tumor while maintaining low doses to normal organs; hence, the indication of radiotherapy for ICC has rapidly expanded [2, 3, 24–26]. SBRT delivers high-dose radiation within a short-term treatment period, and is considered suitable for relatively small tumors (i.e., those less than 5 cm). Tse et al. administered SBRT to patients with ICC at the palliative dose of 24 Gy in 6 fractions (biologic equivalent dose [BED] = 33.6 Gy), and reported an MST of 15 months [24]. Furthermore, Tao et al. recently administered SBRT to 79 patients with unresectable ICC with an escalated dose of 100 Gy to the center of the tumor, while doses to the gross tumor volume and planning target volume were 75 Gy and 45 Gy, respectively [25]. The MST was 33 months with a 3-year overall survival rate of 44%, and they suggested that a high BED (greater than 80.5 Gy) was significantly correlated with better local control and overall survival.^20^

PBT is also useful for intrahepatic tumors because proton beam dose localization can achieve a homogenous dose distribution with simple beam arrangements (such as with 2 or 3 beams). PBT is reportedly suitable as a higher-dose radiotherapy modality for large liver tumors [27]. Makita et al. suggested clinical outcomes and toxicity of PBT for 28 patients with advanced cholangiocarcinoma including 6 patients with ICC. They suggested overall survival, progression survival and local control rate at 1 year was 49.0, 29.5, and 67.7%, respectively, and that better local control was achieved with BED > 70 Gy [28]. Recently, Hong et al. conducted a phase II study of 37 patients with unresectable ICC who were administered 67.5 Gy in 15 fractions (BED: 97.9 Gy), and reported 2-year local control and overall survival rates of 94.1 and 46.5%, respectively [3]. In our current study, the BED of the treatment dose was greater than 96.6 Gy for the curative group, and the 2-year local control and overall survival rates were 71.5 and 52.4%, respectively. Local recurrence was observed in 4 of the 25 patients in the curative group, and all recurrences were marginal to the target region adjacent to the alimentary tract (where full doses were not delivered for safety considerations). Also, decline of CA19–9 at 3 months after PBT was observed in most patients, even with palliative intent in our study. Also, CA19–9 was declined regardless of administration of chemotherapy, and CA19–9 rose in some patients with chemotherapy. These results suggested local control with PBT is effective for control of disease progression. Meanwhile, MST was significantly longer in the patients with maintenance chemotherapy. Therefore, the role of chemotherapy also important with patients who received PBT.

A tumor’s macroscopic subtype is important for determining the optimal surgical procedure because each of the 3 types of ICC has a distinct pathological extension pattern. Intrahepatic recurrence is frequent in MF-type tumors, infiltration along the bile duct is frequent in PI-type tumors, and lymph node metastases are uncommon in patients with IG-type tumors. The postoperative prognosis of patients with these 3 subtypes are also dissimilar, the MSTs of patients with IG-, MF-, and PI-type tumors are reportedly 17–55 months [29, 30], 18–50 months [29–32], and 10–15 months [29, 30], respectively. Although the patients in our cohort had unresectable ICC, the MSTs for those with IG-, MF-, and PI-type tumors were 61, 31, and 9.0 months, respectively, which are comparable to those in patients who undergo surgery though our patient’s number with PI- and IG-type were small. Moreover, our contouring procedure during dose calculation was the same among patients with all 3 subtypes; however, it may be preferable to contour the target regions while taking into account tumor extension characteristics, which differ among these subtypes.

## Conclusion

Our study was limited in that it was retrospective and included a small number of patients; nevertheless, our data suggested that PBT was safe and effective for patients with unresectable ICC, therefore, we consider that curative PBT for ICC is encouraged, though the definitive criteria has not been established yet. Curative-intent treatment and the administration of maintenance chemotherapy were significant predictors of improved survival; furthermore, the macroscopic tumor type also appears to be an important prognostic factor.

## Data Availability

The datasets supporting the conclusions of this article are included within the article.
